# Clinical features of minor hallucinations in different phenotypes of Parkinson’s disease: A cross-sectional study

**DOI:** 10.3389/fneur.2023.1158188

**Published:** 2023-03-24

**Authors:** Yaxi Wang, Dongfeng Li, Yaning Chen, Sha Zhu, Xu Jiang, Yinyin Jiang, Ruxin Gu, Bo Shen, Jun Zhu, Yang Pan, Jun Yan, Li Zhang

**Affiliations:** Department of Geriatric Neurology, The Affiliated Brain Hospital of Nanjing Medical University, Nanjing, China

**Keywords:** Parkinson’s disease, phenotype, tremor dominant, postural instability gait difficulty, minor hallucination, visual illusion

## Abstract

**Background:**

Minor hallucinations (MHs) are the most common psychiatric symptom associated with Parkinson’s disease (PDPsy), but little is known about their characteristics in different motor phenotypes, especially postural instability gait difficulty (PIGD). The aim of this study was to explore the clinical features of MHs in different subtypes of PD.

**Methods:**

In this cross-sectional study, 213 patients with Parkinson’s disease (PD) were recruited, and the data obtained included comprehensive demographics, motor subtypes, clinical scale scores, and MH contents. Motor subtypes were classified as tremor-dominant (TD), PIGD or indeterminate according to Stebbins’ method.

**Results:**

A total of 213 PD patients were included: 90 (42.3%) TD patients, 98 (46.0%) PIGD patients and 25 (11.7%) indeterminate. In total, 70 (32.9%) patients experienced MHs. Compared to patients with the TD phenotype, we found that patients with the PIGD phenotype had more severe motor and nonmotor symptoms. They also had a higher incidence of visual illusions (VIs) and a shorter MH latency.

**Conclusion:**

Our study demonstrated that compared to patients with the TD phenotype, patients with the PIGD phenotype had a higher incidence of MHs, especially VIs, which may lead to a higher incidence of visual hallucinations (VHs). They also had a shorter latency of MHs than patients with the TD phenotype, suggesting an earlier onset of MHs and a worse prognosis.

## Introduction

Parkinson’s disease (PD) is the second most frequent central nervous system degenerative illness (1). Clinically, PD patients are primarily classified into two phenotypes based on motor symptoms: tremor-dominant (TD) and postural instability gait difficulty (PIGD) phenotypes (2). Several studies have shown that the PIGD phenotype has more severe motor and nonmotor symptoms, including hallucinations, than the TD phenotype, which indicates faster disease progression and poorer outcomes (3–6). Hallucinations in PD are usually thought to be a psychiatric complication that occurs during the latter part of the disease (7). Previous studies have mostly focused on the clinical features of well-structured visual hallucinations (VHs) in relation to different phenotypes. As minor hallucinations (MHs) have gradually come into focus, the spectrum of psychosis associated with Parkinson’s disease (PDPsy) has been expanded (8). MHs include presence hallucinations, passage hallucinations and visual illusions (VIs). MHs emerged as a common early nonmotor symptom, possibly before the onset of parkinsonism (9). The risk factors for the early and late onset of MHs need to be explored and identified.

Several studies have shown that VHs are a clinical milestone in PD. VHs suggest a significantly increased risk of death later in disease course and have potential implications for clinical disease staging and prognosis (10, 11). Moreover, the PIGD phenotype is an independent baseline predictor of PD mortality and could have a cumulative effect on mortality if combined with VHs (11). While MH is the most common PDPsy (9) and it has been proposed that MHs are closely related to well-structured VHs, so MHs may be the early predictor of severe VHs (12, 13). There are few studies on the characteristics of MHs in different motor phenotypes. Therefore, the present study aimed to explore the clinical features of MH in different subtypes to identify these at-risk patient groups clinically and seek to have a more positive impact on their prognosis.

## Materials and methods

### Patients

From April 2020 to September 2021, we consecutively enrolled 213 PD patients at the Department of Geriatrics, Brain Hospital of Nanjing Medical University. All patients fullfilled the diagnostic criteria of the Movement Disorders Association (14). They were diagnosed by at least two experienced movement disorder specialists. All patients were followed up for a year to discriminate between dementia with Lewy bodies and PD with dementia (15). Participants were excluded if they had a significant psychiatric history or use of any antipsychotic medication. Those with corrected vision or abnormal vision were also excluded.

Based on the Movement Disorder Society-sponsored revision of the Unified Parkinson’s Disease Rating Scale (MDS-UPDRS) part 1, the presence of MHs was initially screened. According to the following scale: 0 indicates no hallucinations or psychotic behavior, 1 indicates minor hallucinations, 2 indicates formed hallucinations without loss of insight, 3 indicates formed hallucinations with loss of insight, and 4 indicates delusions (16). Patients with an item score of 1 who met the requirement of having MHs on a consistent basis for two months before the study were considered to have isolated MHs and were included in the group.

Using the method proposed by Stebbins (2), those patients were divided into TD, PIGD and intermediate groups. According to the mean scores of the 8 items (UPDRS II Item 16 and UPDRS III Items 20–21) and the mean scores of the 5 items (UPDRS III Items 29–30 and UPDRS II Items 13–15), they were classified as PIGD (ratio ≤ 1), TD (ratio ≥ 1.5), or intermediate (1 < ratio < 1.5).

This study was well reviewed and approved by the ethical committee of the Affiliated Brain Hospital of Nanjing Medical University. All patients signed an independent informed consent form before enrolment.

### Clinical assessment

Patient demographic characteristics were collected by a standardized questionnaire, including age, sex, body mass index (BMI), education, duration of disease, and equivalent daily dose of levodopa (LEDD) (17). The latency of MHs, namely, the time between the first onset of MHs and the onset of motor symptoms, was calculated by subtracting the number of years of MHs from the number of years of motor symptoms. Due to the fact that MHs may precede the onset of motor symptoms, the result may be negative. Based on a previous study, a more detailed questionnaire was used to describe the characteristics of MHs as precisely as possible (18). The severity of motor symptoms was measured by the UPDRS III and Hoehn and Yahr (H-Y) scales, while the Non-Motor Symptom Questionnaire (NMS-Quest) was used to assess nonmotor symptoms. All patients were evaluated with the MoCA for cognitive function and the Hamilton Anxiety Inventory (HAMA) and the Hamilton Depression Inventory (HAMD) for mood. Sleep quality was assessed using the PD Sleep Scale (PDSS) and the Pittsburgh Sleep Quality Index(PSQI).Meanwhile, possible rapid eye movement sleep behavior disorder (pRBD) was further assessed using the Rapid Eye Movement Sleep Behavior Disorder Screening Questionnaire (RBDSQ), and the quality of daily life of these patients was assessed by the Parkinson’s Disease Questionnaire 39 (PDQ39).

### Statistical analysis

This study is primarily a two-sample comparison. The Kolmogorov–Smirnov test was used to test whether the sample was normal. If the values conformed to a normal distribution, Student’s t test was used; otherwise, the Mann–Whitney U test was used. Between the PD-TD and PD-PIGD groups, Student’s t test was used in the analysis of age and BMI, while disease duration; H-Y stage; UPDRS III scores; LEDD; and UPDRS I, UPDRS II, UPDRS IV, HAMA, HAMD, NMS-Quest, PSQI, PDSS, MoCA, RBDSQ and PDQ39 scores were analyzed by Mann–Whitney U test. Between MH-TD and MH-PIGD groups, Student’s t test was applied in the analysis of age, LEDD, HAMA, HAMD, NMS-Quest, PSQI and PDQ39 scores, while BMI; disease duration; H-Y stage; and UPDRS III, UPDRS I, UPDRS II, UPDRS IV, PDSS, MoCA and RBDSQ scores were analyzed by Mann–Whitney U test. All consecutive variables are shown as the mean ± standard deviation (SD). Categorical variables such as male sex, education, the prevalence of MHs and the contents of MHs are expressed as percentages and analyzed by chi-square test and Pearson’s and Fisher’s exact test. Correlations between the latency of MHs and clinical characteristics were analyzed by Spearman’s rank correlation coefficient (r_s_). Data analysis was performed using SPSS 26.0.There was a significant difference when values of *p* < 0.05.

## Results

### Demographic characteristics of patients

**Figure 1:**
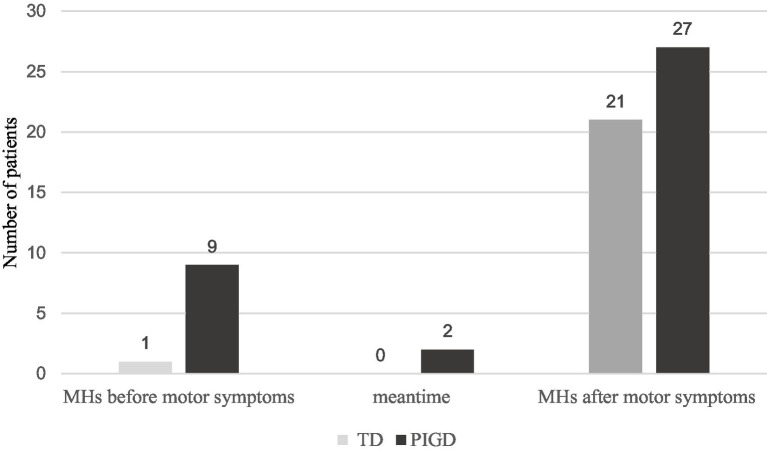
The number of patients with MHs onsets between the TD and PIGD groups in the cohort. There were 70 patients with MHs in our study. Among them were 22 (31.4%) TD patients and 38 (54.3%) PIGD patients. In the TD group, it included 1 patient who experienced MHs before motor symptoms and 21 patients who experienced MHs after motor symptoms. In the PIGD group, 9 patients experienced MHs before motor symptoms and 27 patients experienced MHs after motor symptoms. The remaining 2 patients experienced MHs when motor symptoms appeared.

A total of 213 patients with PD were recruited for this study, including 90 (42.3%) TD patients, 98 (46.0%) PIGD patients and 25 (11.7%) indeterminate patients. Seventy (32.9%) patients experienced MHs. Among them were 22 (31.4%) TD patients, 38 (54.3%) PIGD patients and 10 (14.3%) indeterminate patients. In the TD group, it included 1 patient who experienced MHs before motor symptoms and 21 patients who experienced MHs after motor symptoms. In the PIGD group, 9 patients experienced MHs before motor symptoms and 27 patients experienced MHs after motor symptoms. The remaining 2 patients experienced MHs when motor symptoms appeared. The demographic characteristics between the TD and PIGD groups are summarized in Table 1 and Figure 1. In total patients, there were no differences in age, sex, BMI, education, disease duration or UPDRS III scores. However, there were significant differences between H-Y stage, LEDD and prevalence of MHs in the two groups. In MHs, no significant differences were found between the two groups.

**Table 1:** Demographic characteristics of patients.

	Total			MH		
	TD	PIGD	*p*	TD	PIGD	*p*
Number	90	98		22	38	
Age (years)	66.41 ± 8.48	67.43 ± 10.01	0.456	66.45 ± 8.13	68.39 ± 9.91	0.440
Male (%)	50 (55.6%)	45 (45.9%)	0.187	14 (63.6%)	19 (50%)	0.306
BMI	23.63 ± 3.43	23.62 ± 3.35	0.981	23.44 ± 3.01	22.66 ± 3.15	0.505
Education (%)			0.265			0.133
Illiteracy	12 (13.3%)	13 (13.3%)		2 (9.1%)	4 (10.5%)	
Primary school	18 (20.0%)	10 (10.2%)		7 (31.8%)	3 (7.9%)	
Middle school	45 (50.0%)	53 (54.1%)		10 (45.5%)	24 (63.2%)	
College or above	15 (16.7%)	22 (22.4%)		3 (13.6%)	7 (18.4%)	
H-Y stage	2.20 ± 0.66	2.59 ± 0.57	**<0.001**	2.50 ± 0.56	2.51 ± 0.62	0.722
Disease duration(years)	5.58 ± 4.22	5.70 ± 4.19	0.782	7.40 ± 4.06	5.84 ± 4.26	0.106
UPDRSIII	31.06 ± 16.13	30.47 ± 12.89	0.945	35.55 ± 17.97	27.84 ± 11.65	0.125
LEDD (mg)	383.55 ± 377.74	546.94 ± 392.55	**0.002**	446.69 ± 264.99	561.98 ± 345.64	0.182
MH(%)	22 (24.4%)	38 (38.8%)	**0.035**	/	/	

Data are presented as the mean ± SD, *n* (%). Bold value is statistically significant (*p* < 0.05). MH, minor hallucination; TD, tremor dominant; PIGD, postural instability and gait difficulty; BMI, body mass index; H-Y, Hoehn and Yahr; UPDRS III, Unified Parkinson’s Disease Rating Scale part 3; LEDD, Levodopa equivalent daily dose.

### Clinical scale data

Among all patients, clinical scale data including UPDRS IV, MoCA, PSQI, and PDSS scores, were similar between the two groups. Compared with the TD group, UPDRS I, UPDRS II, HAMA, HAMD, NMS-Quest, RBDSQ and PDQ39 scores were significantly higher in the PIGD group. In patients with MHs, significant differences in UPDRS II, HAMA, MoCA, PSQI, PDSS, and PDQ39 scores were also observed between the two groups, but there were no differences in other scales. All data are presented in Table 2.

**Table 2:** Comparison of clinical scale data between the TD and PIGD patients.

	Total			MH		
	TD	PIGD	*p*	TD	PIGD	*p*
UPDRS I	2.23 ± 2.29	2.98 ± 2.11	**0.004**	2.68 ± 1.62	4.03 ± 2.05	**0.012**
UPDRSII	12.03 ± 6.10	13.53 ± 5.44	**0.047**	15.05 ± 7.19	13.95 ± 4.35	0.860
UPDRSIV	1.30 ± 2.15	2.09 ± 2.75	0.050	2.09 ± 2.49	2.26 ± 2.84	0.987
HAMA	6.01 ± 4.48	8.01 ± 4.74	**0.002**	7.50 ± 4.64	9.68 ± 4.54	0.080
HAMD	7.08 ± 4.85	9.60 ± 5.13	**<0.001**	8.23 ± 4.20	11.26 ± 5.06	**0.021**
MoCA	24.20 ± 5.04	23.34 ± 5.93	0.333	24.36 ± 4.32	23.42 ± 5.75	0.700
NMS-Quest	10.36 ± 5.21	13.89 ± 5.15	**<0.001**	13.18 ± 5.60	17.26 ± 4.40	**0.003**
RBDSQ	2.17 ± 2.04	3.86 ± 2.81	**<0.001**	2.50 ± 2.52	5.16 ± 2.54	**<0.001**
PSQI	6.80 ± 5.07	7.31 ± 4.56	0.361	7.77 ± 4.61	7.97 ± 4.75	0.874
PDSS	109.48 ± 27.62	108.63 ± 26.54	0.697	91.71 ± 27.09	100.52 ± 26.39	0.182
PDQ39	39.0.12 ± 25.92	53.71 ± 26.34	**<0.001**	46.91 ± 29.56	60.76 ± 26.47	0.066

Data are shown as mean ± SD; Bold data are statistically significant (*p* < 0.05). MH, minor hallucination; TD, tremor dominant; PIGD, postural instability and gait difficulty; UPDRS I, Unified Parkinson’s Disease Rating Scale part1; UPDRS II, Unified Parkinson’s Disease Rating Scale part 2; UPDRSIV, Unified Parkinson’s Disease Rating Scale part 4; HAMA, Hamilton Anxiety Rating Scale; HAMD, Hamilton Depression Rating Scale; MoCA, Montreal Cognitive Assessment; NMS-Quest, Non-Motor Symptoms Questionnaire; RBDSQ, the REM sleep behavior disorder Sreening Questionnaire; PSQI, Pittsburgh Sleep Quality Index; PDSS, Parkinson’s Disease Sleep Scale; PDQ39, Parkinson’s Disease Questionnaire-39.

### Characteristics of minor hallucinations

In our research, we found that the MH latency was significantly shorter in the PIGD group than in TD group (6.18 ± 3.93 vs. 2.57 ± 5.07, *p* = 0.005). Additionally, passage hallucinations (59.1%) were more common in the TD group, while in the PIGD group, visual illusions (76.3%) were more common. There was a significant difference in the prevalence of visual illusions between the two groups (*p* = 0.016). Regarding the MH specifics, the study found that MHs could occur at any time, but were more likely to occur during the daytime in both groups. MHs in both groups tended to appear suddenly and last only a few seconds. However, compared to the TD group, MHs were more likely to occur when the light was bright in the PIGD group (47.4%). The frequency of MHs was less than once a week in both groups, and the hallucinated images was normal in size and monochrome. There were no significant differences between the two groups in the seven entries. The specific results are shown in Table 3.

**Table 3:** Comparison of minor hallucinations between the TD and PIGD patients.

	All	TD	PIGD	*p*
Number	60	22	38	
Latency(years)	3.89 ± 4.97	6.18 ± 3.93	2.57 ± 5.07	**0.005**
Subtype				
Presence hallucination	23 (38.3%)	7 (31.8%)	16 (42.1%)	0.430
Passage hallucination	30 (50.0%)	13 (59.1%)	17 (44.7%)	0.284
Visual illusion	39 (65.0%)	10 (45.5%)	29 (76.3%)	**0.016**
Appearance time				0.612
Daytime	40 (66.7%)	14 (63.6%)	26 (68.4%)	
Nighttime	10 (16.7%)	5 (22.7%)	5 (13.2%)	
Both	10 (16.7%)	3 (13.6%)	7 (18.4%)	
Lighting				0.180
Bright	27 (45.0%)	9 (40.9%)	18 (47.4%)	
Dim	30 (50.0%)	13 (59.1%)	17 (44.7%)	
Both	3 (5.0%)	0 (0.0%)	3 (7.9%)	
Form of occurrence				1.000
Sudden	59 (98.3%)	22 (100.0%)	37 (97.4%)	
Gradual	1 (1.7%)	0 (0.0%)	1 (2.6%)	
Lasting time				1.000
Seconds	47 (78.3%)	17 (77.3%)	30 (78.9%)	
Minutes	13 (21.3%)	5 (22.7%)	8 (21.1%)	
Frequency				0.869
Daily	19 (31.7%)	7 (31.8%)	12 (31.6%)	
≥1/week	13 (21.7%)	4 (18.2%)	9 (23.7%)	
<1/week	28 (46.7%)	11 (50.0%)	17 (44.7%)	
Size				0.323
Normal	55 (91.7%)	20 (90.9%)	35 (92.1%)	
Magnified	1 (1.7%)	1 (4.5%)	0 (0.0%)	
Miniaturized	4 (6.7%)	1 (4.5%)	3 (7.9%)	
Color				0.583
Monochrome	33 (55.0%)	14 (63.6%)	19 (50.0%)	
Colorized	24 (40.0%)	7 (31.8%)	17 (44.7%)	
Both	3 (5.0%)	1 (4.5%)	2 (5.3%)	

Values are shown as *n* (%). Bold value is statistically significant (*p* < 0.05). TD, tremor dominant; PIGD, postural instability and gait difficulty.

### Correlations between the presence of visual illusion and the clinical characteristics

The outcomes of Spearman’s correlation analysis between the latency of MHs and other clinical assessment scales are summarized in Table 4. MH latency was negatively correlated with PD subtype(r_s_ = −0.369, *p* = 0.004) and RBDSQ (r_s_ = −0.026, *p* = 0.044). However, there was no correlation between latency and NMS-Quest, PDSS,PSQI, HAMA, HAMD, and MoCA scores.

**Table 4:** Correlations between latency and clinical characteristics.

Variable	rs	*p*
Subtype	−0.369	**0.004**
NMS-Quest	−0.071	0.588
PDSS	−0.077	0.556
PSQI	−0.083	0.529
RBDSQ	−0.026	**0.044**
HAMA	−0.106	0.421
HAMD	−0.087	0.510
MoCA	−0.110	0.403

Significant data are highlighted in bold (*p* < 0.05). r_s_, Spearman’s rank correlation coefficient; NMS-Quest: Non-Motor Symptoms Questionnaire; PDSS, Parkinson’s Disease Sleep Scale; PSQI, Pittsburgh Sleep Quality Index; RBDSQ, the REM sleep behavior disorder Sreening Questionnaire; HAMA, Hamilton Anxiety Rating Scale; HAMD, Hamilton Depression Rating Scale; MoCA: Montreal Cognitive Assessment.

## Discussion

This study recruited 213 PD patients to investigate the clinical features of the different motor phenotypes and the characteristics of MHs. Compared to patients with the TD phenotype, we found that patients with the PIGD phenotype had more severe motor and nonmotor symptoms, poorer quality of life, a shorter MH latency and a higher incidence of VIs. In addition, MH latency was correlated with high RBDSQ scores. To our knowledge, this is the first paper to explore the characteristics of MHs in different motor subtypes of PD patients.

In this study, PIGD was observed in 46.0% of PD patients, which within in the range described in prior research (approximately 18–56%) (5, 19–21). Compared to patients with the TD phenotype, patients with the PIGD phenotype had more severe affective disorders and sleep disturbances, including depression and high RBDSQ scores which may indicate possible rapid eye movement (REM) sleep behavior disorder (RBD). These foundings were consistent with other reports (5, 22–24). These results may be related to norepinephrine insufficiency in the peripheral and central nervous systems in the PIGD phenotype (25). A recent study (26) suggests that cholinergic system alterations could also be associated with PIGD. However, we did not find poorer cognitive performance in the PIGD group than in the TD group, which was different from previous results (27, 28). We speculate that this may be due to the use of different measurement tools. Additionally, the majority of the patients in our research were in the early stages of the disease which may be an additional explanation for our differing results (29). Taken together, the PIGD phenotype probably represents a significantly more ‘aggressive’ PD phenotype, which indicates more diffuse and advanced neurodegeneration (30).

Ffytche, D. H. et al. hypothesized that PDPsy may be a continuous and progressive process that begins with MHs, and develops into large hallucinations with insight, gradually losing insight and eventually turning into delusions (31). In our PD population, patients with the PIGD phenotype had a higher incidence and shorter latency of MHs than patients with the TD phenotype. These findings suggest that, compared to patients with the TD phenotype, patients with the PIGD phenotype are more likely to suffer from MHs and these MHs appear earlier. There are several possible reasons for these results. First, compared to the TD phenotype, cortical and subcortical engagement were already present to a greater extent at the onset of the PIGD phenotype (3). Impaired frontal lobe function has been shown to be associated with the development of PIGD phenotype (32, 33). Structural and functional abnormalities of the frontal cortex, including the prefrontal cortex and orbitofrontal cortex, have also been found in PD patients with VHs (34, 35). Recently, Zhang, Y. et al. pointed out that impaired frontal lobe function is an independent predictor of MHs. Therefore, impaired frontal lobe function may play an important role in PIGD and MH comorbiditiesc. In addition, while the PIGD phenotype may not be a direct cause of psychosis including hallucinations, several dominant risk factors for psychosis, such as high RBDSQ scores, are more prevalent in PIGD individuals than in TD patients (6, 12, 36), so these risk factors may indirectly contribute to the development of psychosis in the PIGD phenotype.

Our study also found that the latency of MHs was inversely correlated with RBDSQ scores. Numerous studies have demonstrated a significant association between the presence of RBD and the occurrence of hallucinations in PD (12, 37, 38). It may be associated with cholinergic dysfunction involved in the pathophysiology of hallucinations (36, 39, 40). Archibald et al. (41) reported that the presence of RBD is associated with MHs, especially the sense of presence. Given these findings, MHs, as the most common hallucinations among PD patients, even appear in the premotor phase (9, 42), deserving more attention when they appear in different motor phenotypes.

In addition, we found that patients with the PIGD phenotype had a higher incidence of VIs than those with the TD phenotype. The link between hallucations and altered dream experiences, intrusion of REM sleep-related imagery into awakefulness, and RBD may explain this result (43, 44). Vivid dreams, known to be associated with RBD, predispose individuals to hallucination/illusion formation (44). Furthermore, it has even been shown that RBD is an independent predictor of MHs in PD patients without dementia (42). In our study, the RBDSQ scores was higher in patients with the PIGD phenotype than in those with the TD phenotype, which may indicating possible RBD and lead to the higher incidence of VIs in the PIGD phenotype in this population. The proposed hypothesis observed that VIs and VHs were continuous situations with the same underlying processes (8, 45). Clinically, VH is described as perceptions that occur in the absence of existing stimuli, while VI is characterized as misleading perceptions of real stimuli. Although they differ in definition, their neurologically related factors overlap considerably (45). According to a structural neuroimaging study, both symptoms have been linked to abnormalities of the temporoparietal cortices, which assist visual spatial processing (46). Therefore, we speculate that the high incidence of VIs in patients with the PIGD phenotype may lead to a higher incidence of VHs. VHs, as a clinical milestone in PD suggest a significantly increased risk of death later in the disease course (11). Frequent VHs could cause distress to both patients and caregivers, and increase the difficulty of daily care (47). Therefore, identifying these at-risk patient groups earlier may be clinically useful since treating such individuals may improve their quality of life. However, there has been little research on VIs in PD, and thus far, few studies have addressed neuropathological mechanisms and brain changes that may be specifically related to VIs or VHs. Given this, further studies are necessary to focus on the evolution of VIs and explore the relationship between VIs and VHs.

Our study had some limitations. First, there may be bias in the patient’s report of MHs depending on the patient’s recollection, even though the presence of MHs was assessed by at least two experienced assessors. Therefore, it may be meaningful to use a combination of independent recall tools and semistructured interviews in the MH survey. Second, this study is a cross-sectional study and lacks a longitudinal study to observe the evolution of VIs. This cohort will be followed up for many years to observe the evolution of their MHs, especially VIs. Finally, MHs tend to be clinically overlooked due to their concealment (48). Patients who have hallucinations may be ashamed to tell their doctors about them. Therefore, many of them avoid answering this question (49). All of these reasons may lead to MHs not being recorded. Moreover, LEDD doses were higher in the PIGD subgroup in our study, which may lead to an increased incidence of MHs in the PIGD subgroup. However, increasing evidence suggested that the appearance of MHs represented the more aggressive PD, regardless of levodopa doses (18, 50). Therefore, LEDs should be used as potential confounders for additional analysis in subsequent studies.

## Conclusion

Compared to patients with the TD phenotype, patients with the PIGD phenotype had more severe motor and nonmotor symptoms. Additionally, patients with the PIGD phenotype had a higher incidence of MHs, especially VIs, which may lead to a higher incidence of VHs. They had a shorter MH latency than those with the TD phenotype, suggesting an earlier onset of MHs and a worse prognosis. Based on these findings, early identification and intervention are important for these groups.

## Data availability statement

The raw data supporting the conclusions of this article will be made available by the authors, without undue reservation.

## Ethics statement

The studies involving human participants were reviewed and approved by Ethics Committee of the Affiliated Brain Hospital of Nanjing Medical University. The patients/participants provided their written informed consent to participate in this study.

## Author contributions

LZ and YW conceived and designed the study. LZ, JY, and XJ obtained the funding. RG, DL, YC, SZ, YJ, RG, XJ, BS, and JZ collected the data. YW, DL, and YP conducted the data analysis. YW drafted the manuscript. All authors contributed to the article and approved the submitted version.

## Funding

This study was supported by National Natural Science Foundation of China (82171249 and 82101332), Special Funds of the Jiangsu Provincial Key Research and Development Program (BE2019612), Jiangsu Provincial Cadre Health Projects (BJ20005), Jiangsu Provincial Elderly Health Research Project (LD2021013 and LR2021018), Nanjing Rehabilitation Medicine Center Project, Nanjing Industrial and Information Development Special Fund Project, Nanjing Medical Science and Technology Development Foundation (QRX17026), Nanjing Medical University School Fund Project (NMUB20210223).

## Conflict of interest

The authors declare that the research was conducted in the absence of any commercial or financial relationships that could be construed as a potential conflict of interest.

## Publisher’s note

All claims expressed in this article are solely those of the authors and do not necessarily represent those of their affiliated organizations, or those of the publisher, the editors and the reviewers. Any product that may be evaluated in this article, or claim that may be made by its manufacturer, is not guaranteed or endorsed by the publisher.
